# Development and Clinical Application of a Recombinase Polymerase Amplification-Lateral Flow Strip Assay for Detection of Carbapenem-Resistant *Acinetobacter baumannii*

**DOI:** 10.3389/fcimb.2022.876552

**Published:** 2022-05-11

**Authors:** Lei Wang, Dunpo Sun, Li Chen, Ping Zhou, Kun Wang, Fang Wang, Xingqi Lei, Yan Wang, Yingzhi Lu, Guanhong Huang, Xuzhu Gao

**Affiliations:** ^1^ Department of Central Laboratory, Department of Laboratory Medicine, the Second People’s Hospital of Lianyungang City (Cancer Hospital of Lianyungang), Lianyungang, China; ^2^ School of Biotechnology, Jiangsu University of Science and Technology, Zhenjiang, China; ^3^ Department of Acupuncture and Moxibustion, Lianyungang Affiliated Hospital of Nanjing University of Traditional Chinese Medicine, Lianyungang, China

**Keywords:** carbapenem-resistant *Acinetobacter baumannii*, recombinase polymerase amplification, lateral flow strip, *bla*_OXA-51_ gene, *bla*_OXA-23_ gene

## Abstract

*Acinetobacter baumannii* is a worldwide, primary cause of respiratory tract infections, septicemia, urinary apparatus infections, and secondary meningitis. It can be fatal. Rapid and accurate detection methods are needed to control the spread of carbapenem-resistant *A. baumannii* (CRAB). Current molecular diagnostic methods are limited and not suitable for on-site detection. In this study, an isothermal detection method using recombinase polymerase amplification (RPA) combined with a lateral flow strip (LFS) was developed to target the *bla*_OXA-51_ and *bla*_OXA-23_ genes of *A*. *baumannii*. The reaction was completed in about 40 min at 37°C. This method can also effectively distinguish *A. baumannii* and CRAB. The limit of detection of 10^0^-10^1^ CFU/reaction was equal to that of other detection methods. The detection accuracy was equal to that of the qPCR method with the use of clinical samples. The RPA-LFS assay is portable, rapid, and accurate and could replace existing detection methods for on-site detection of *A*. *baumannii* and CRAB.

## Introduction

*Acinetobacter baumannii* is a common pathogen of nosocomial infections (Chung et al., 2011; Mirshekar et al., 2018). The 2020 China Antimicrobial Surveillance Network reported that number of infections caused by *A. baumannii* continues to increase, accounting for 17.07% of lower respiratory tract infections and a mortality rate of 35% (Shaheen et al., 2017). *A. baumannii* is categorized by the World Health Organization as among the most dangerous bacteria (Song et al., 2016; Tacconelli et al., 2018). In addition, *A. baumannii* is resistant to several antibiotics and, thus, has attracted the attention of microbiologists and doctors (Tekin et al., 2014). *A. baumannii* is a major cause of respiratory tract infections, septicemia, urinary apparatus infections, and secondary meningitis (Ranjbar et al., 2020). *A. baumannii* is widely distributed and can survive for long periods in hospital settings, thereby posing a serious threat to patients in the intensive care unit (Perez et al., 2007). Notably, carbapenem-resistant *A. baumannii* (CRAB) continues to rapidly spread globally (Villegas and Hartstein, 2003).

*A. baumannii* is categorized into five associated subgroups based on the production of oxacillinase (OXA): OXA-51, which is intrinsic, and OXA-143, OXA-58, OXA-40, and OXA-23, which are acquired (Woodford et al., 2006; Lee et al., 2009). CARB is resistant to various antimicrobial agents, mainly due to the production of OXA and metallo-β-lactamase (Azimi et al., 2013; Ei et al., 2019). The most common CRAB isolates produce OXA-23 carbapenemase (Chen et al., 2013). The *bla*_OXA-51_ gene is an established marker for detection of *A. baumannii*, while the *bla*_OXA-23_ gene is the most frequent carbapenemase gene detected in CRAB isolates (Turton et al., 2006; Chen et al., 2013; Djahmi et al., 2014; Karampatakis et al., 2017).

Rapid detection of CRAB can facilitate early treatment and minimize the severity of infection. Several diagnostic methods have been reported for the detection of *A. baumannii*, including loop-mediated isothermal amplification (LAMP), polymerase chain reaction (PCR), quantitative PCR (qPCR), and culture-based methods (Huang et al., 2012; Wang et al., 2014; Mu et al., 2016; Nirwati et al., 2018). Although these methods have unique advantages, all are limited by time requirements, low sensitivity, need for thermocycling equipment, and dependence on trained personnel. These drawbacks may inhibit the application of these methods in the field for everyday monitoring. To combat the extensive spread of CRAB, it is important to establish an on-site diagnostic method that is simple, rapid, accurate, and inexpensive.

Recombinase polymerase amplification (RPA) is an isothermal DNA amplification technology first reported in 2006 and widely used in recent years (Piepenburg et al., 2006). The RPA system relies on recombinase (UvsX and UvsY), single-stranded binding protein (gp32), and strand-displacing DNA polymerase (Bsu) for nucleic acid amplification. The reaction is completed in about 30 min at a constant temperature of 25–42°C, usually at 37°C, (Dong et al., 2020; Wang et al., 2020; Wang et al., 2022). The amplification products of RPA can be detected using gel electrophoresis, a fluorescence detector, and a lateral flow strip (LFS) (Khater et al., 2019; Ma et al., 2021; Wang et al., 2022). However, the sensitivity of gel electrophoresis and fluorescence detection is limited outside of a laboratory. On the contrary, a LFS is suitable for simple testing and the detection results can be analyzed visually without the need for complex thermocycling equipment and trained personnel (Li et al., 2020). The RPA-LFS assay can achieve a quick response time and good accuracy when used as a diagnostic test for a variety of infectious diseases (Dong et al., 2020; Yang et al., 2021).

In this study, an accurate RPA-LFS assay for detection of CRAB was established by designing specific primers and probes for detection of the *bla*_OXA-51_ and *bla*_OXA-23_ genes. This is the first report of the detection of *A. baumannii* in sputum with the use of the RPA-LFS assay and to distinguish CRAB *via* detection of *A. baumannii* (*bla*_OXA-51 without OXA-23_) and *A. baumannii* (*bla*_OXA-51 and OXA-23_). This method can confirm infection of CRAB, but not common *A. baumannii*, to facilitate early treatment and prevent severe illness.

## Materials and Methods

### Collection of Samples and DNA Extraction

*A. baumannii* and *Candida albicans* were obtained from the American Type Culture Collection (Manassas, VA, USA). In addition, isolates of *A. baumannii* (*bla*_OXA-51 without OXA-23_), isolates of *A. baumannii* (*bla*_OXA-51 and OXA-23_) strains, isolates of other *Acinetobacter* species, and isolates of common infectious pathogens were provided by The Second People’s Hospital of Lianyungang (Lianyungang, China). The sputum isolates of *A. baumannii* were collected from patients aged 20–50 years and hospitalised for at least one week. Swab of the wound, sputum, and urine clinical samples were obtained from the ICU hospitalized patients with clinically suspected multi-resistant infections. Information of all strains and samples are listed in **Table 1**. The identities of all isolates were confirmed by 16S rRNA PCR and qPCR (Huang et al., 2012; Misbah et al., 2015). All strains were cultured in Luria–Bertani broth at 37°C while shaking at 200 rpm. Cultures of 10^7^ colony-forming units (CFU)/μL were boiled at 100°C for 10 min as DNA templates. The DNA templates were confirmed as originating from the respective pathogens by qPCR as described previously. The PCR products amplified with 16S rRNA primers were sequenced using the first generation sequencing techniques by ABI 3730XL Genetic Sequencer, and confirmed by General Biosystems Co. Ltd. (Anhui, China).

**Table 1:** Bacteria strains used in this study.

Species	Source	Strain designation	Number of samples
*Acinetobacter baumannii*	Reference strain	ATCC 19606	1
*Acinetobacter baumannii* (*bla*_OXA-51 without OXA-23_)	Sputum isolated strain	#1 #2 #3 #4 #5 #6 #7 #8 #9 #10	10
*Acinetobacter baumannii* (*bla*_OXA-51 and OXA-23_)	Sputum isolated strain	#1 #2 #3 #4 #5 #6 #7 #8 #9 #10	10
Clinical samples	Sputum	N/A	78
Clinical samples	Swab of the wound	N/A	49
Clinical samples	Urine	N/A	86
*Acinetobacter calcoaceticus*	Sputum isolated strain	N/A	1
*Acinetobacter lwoffi*	Sputum isolated strain	N/A	1
*Acinetobacter haemolytius*	Sputum isolated strain	N/A	1
*Acinetobacter junii*	Sputum isolated strain	N/A	1
*Acinetobacter johnsonii*	Sputum isolated strain	N/A	1
*Candida albicans*	Reference strain	ATCC 10231	1
*Enterobacter cloacae*	Sputum isolated strain	N/A	1
*Enterococcus faecium*	Sputum isolated strain	N/A	1
*Escherichia coli* O157	Sputum isolated strain	N/A	1
*Mycobacterium tuberculosis* H37Ra	Sputum isolated strain	N/A	1
*Pseudomonas aeruginosa*	Sputum isolated strain	N/A	1
*Staphylococcus aureus*	Sputum isolated strain	N/A	1
*Staphylococcus capitis*	Sputum isolated strain	N/A	1
*Staphylococcus epidermidis*	Sputum isolated strain	N/A	1
*Staphylococcus haemolyticus*	Sputum isolated strain	N/A	1
*Staphylococcus hominis*	Sputum isolated strain	N/A	1
*Staphylococcus saprophytics*	Sputum isolated strain	N/A	1
*Staphylococcus wameri*	Sputum isolated strain	N/A	1
*Stenotrophomonas maltophilia*	Sputum isolated strain	N/A	1
*Streptococcus pneumonia*	Sputum isolated strain	N/A	1
*Viridans streptococci*	Sputum isolated strain	N/A	1
*Klebsiella pneumoniae*	Sputum isolated strain	N/A	1

ATCC, American Type Culture Collection, Manassas, VA, USA.

### Design of Primers and Probes

Primers and probes for the RPA-LFS assay were designed to target the sequences of the *bla*_OXA-51_ gene (National Center for Biotechnology Information [NCBI] reference sequence: CP043953.1) and *bla*_OXA-23_ gene (NCBI reference sequence: NG_049525.1). Forward and reverse primers were designed with the Primer-Basic Local Alignment Search Tool (BLAST) (https://www.ncbi.nlm.nih.gov/tools/primer-blast/). The key parameter settings were as follows: minimum and maximum product sizes, 50 and 250 bp, respectively; minimum and maximum primer sizes, 31 and 35 nt, respectively; and minimum and maximum guanine-cytosine (GC) content, 20% and 70%, respectively. Other parameters were applied at default settings. The RPA amplification products were analyzed on a 1.5% agarose gel.

The probes were designed using Primer Premier 5 software (Premier Biosoft, Palo Alto, CA, USA) based on the sequences of regions defined by the selected primer pairs. The minimum and maximum sizes of the probes were 45 and 50 bp, the minimum and maximum melting temperatures (*Tm*) were 50°C and 100°C, and the minimum and maximum GC contents were 20% and 80%, respectively. In addition, if the probes and primers had three consecutive matching bases, the probes were mutated to avoid false-positive results.

### RPA-LFS Procedure

RPA reactions were conducted in accordance with the manufacturer’s instructions of the Twist Amp^®^ DNA Amplification nfo Kit (TwistDx Ltd., Maidenhead, UK). Each 25-μL reaction mixture contained 1.05 μL of each primer (10 μM), 0.3 μL of the probe (10 μM), 1.0 μL of the template, and other standard reaction components. Primers and probes were synthesized by General Biosystems Co. Ltd. To initiate the reaction, 1.25 μL of magnesium acetate (280 mM) were added. The reaction mixture was incubated for 30 min at 37°C. Then, 5 μL of the amplification product were spotted on the LFS (Ustar Biotechnologies Ltd., Hangzhou, China). The LFS was composed of a sample pad, conjugate pad (soaked with mouse-originated AuNP-tagged anti-FITC antibody), test line (coated with streptavidin), control line (coated with anti-mouse antibody), and absorbent pad that lined up through the solvent migration route. The RPA amplification product was added to the sample pad of the LFS and the stick of the LFS was inserted into 100 μL of the solvent (Ustar Biotechnologies Ltd.) for about 10 min until the test and control lines were visualized. Totally, the reaction is completed in 30 min isothermally at 37°C and the result can be observed on a LFS in 10 min.

To determine the suitability of the RPA-LFS assay to specifically detect *bla*_OXA-51_ and *bla*_OXA-23_, 20 A*. baumannii* (*bla*_OXA-51 without OXA-23_) and *A. baumannii* (*bla*_OXA-51 without OXA-23_) isolates from sputum were used as templates (10^7^ CFU). The specificity of the primer-probe set *bla*_OXA-51_-F3/P/R2B was tested with the RPA-LFS assay using different sample templates of microbes isolated from sputum. Reference strains of *C. albicans* and *A. baumannii* were also tested (**Table 1**). The amount of the templates was set at 10^7^ CFU. The LOD was first determined with pure *A. baumannii* (*bla*_OXA-51 without OXA-23_). The amount of template was tested at 10^7^–10^0^ CFU/μL (1 μL for each reaction).

### qPCR Procedure

qPCR detection procedure of *A. baumannii* and CRAB were performed as previously reported (Martín-Peña et al., 2013). The primers *bla*_OXA-51_-qF (5’-GCA ACC ACC ACA GAA G-3’) and *bla*_OXA-51_-qR (5’-TCC AAT ACG ACG AGC T-3’) were designed to detect *A. baumannii*, while the primers *bla*_OXA-23_-qF (5’-ATC GGA TTG GAG AAC C-3’) and *bla*_OXA-23_-qR (5’-CCT GAT AGA CTG GGA CT-3’) were used to detect CRAB.

## Results

### Design of RPA Primers and Probes for CRAB Detection

**Figure 1:**
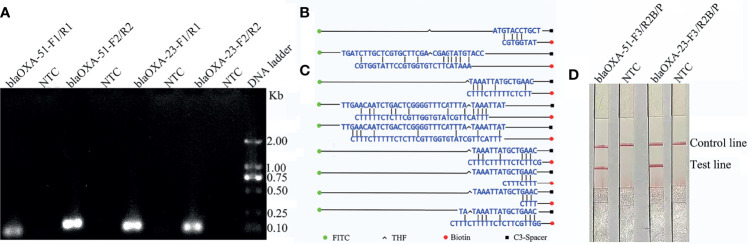
Screening of the primer-probe sets. **(A)** The RPA results of the four different primer sets targeting *bla*_OXA-51_ and *bla*_OXA-23_. The name of each set is shown at the top of each lane. A NTC was used in the reactions. All reactions were performed at 37°C for 30 min. The image represents the results of three independent experiments. **(B, C)** Pairing analysis and sequence modifications of the primer-probe sets for detection of *bla*_OXA-51_ and *bla*_OXA-23_ with Primer Premier 5 software. Relevant DNA bases of the probes and primers were excluded. The DNA strands are shown as horizontal lines and matching bases are indicated with vertical lines. Molecular markers are listed under the **(C)**. **(D)** Effectiveness of the primer-probe sets for the RPA-LFS assay. The name of each set is shown at the top of each lane. A NTC was used in the reactions. The positions of the test and control lines are indicated on the right. All reactions were performed at 37°C for 30 min. The images represent the results of three independent experiments.

The results showed that the four primer pairs produced no obvious primer-dependent artifacts when the DNA template was excluded (**Figure 1A**). The primer sets *bla*_OXA-51_-F2 and *bla*_OXA-23_-F2 were used to design the probes as 5’ end. Possible pairing between the probe and reverse primer of *bla*_OXA-51_ was analyzed to identify the cause of false-positive signals. False-positive signals could result if the probe and reverse primer share three or five consecutive matching bases (**Figure 1B**). To disrupt consecutive matching, two bases were substituted on the probe (C < G and T < G). Likewise, false-positive signals could result if the probe and reverse primer of *bla*_OXA-23_ shared three or four consecutive matching bases (**Figure 1C**), thus five bases were substituted on the probe (A < T, A < C, T < C, A < T, and A < G). Screening two more primers in front of probes according to the primer design principle, they were named as *bla*_OXA-51_-F3 and *bla*_OXA-23_-F3. The sequences of the primers and modified probes are listed in **Table 2**, where base substitutions are highlighted in red. The use of these two modified primer-probe sets for the RPA-LFS assay prevented false-positive signals with no DNA template control (NTC). Since the results indicate that the amplifications were not affected (**Figure 1D**), the modified primer-probe sets were used in this study.

**Table 2:** Primers and probes used in this study.

Primers/Probes	Primer Sequences	Size (bp)	Reaction name	Targeting area	Production Size(bp)
*bla*_OXA-51-_F1	CTATTCCGGTTTATCAAGATTTAGCTCGTCG	31	RPA	2228255..2228343	89
*bla*_OXA-51-_R1	ATCTGCATTGCCATAACCAACACGCTTCACT	31			
bla_OXA-51-_F2	AGCTATGGTAATGATCTTGCTCGTGCTTCGA	31		2228062..2222178	117
bla_OXA-51-_R2	AAATACTTCTGTGGTGGTTGCCTTATGGTGC	31			
bla_OXA-23-_F1	TTTAATGGTCCTACCAACCAGAAATTATCAACCT	35		411..513	103
bla_OXA-23-_R1	GTATCGGTCTTGATCTCATGCAAAAAGAAGTAAAA	34			
bla_OXA-23-_F2	CTTGAACAATCTGACTCGGGGTTTCATTTATT	35		820..910	91
bla_OXA-23-_R2	TTTACTTGCTATGTGGTTGCTTCTCTTTTTCTTTC	32			
bla_OXA-51-_P	FITC-AGCTATGGTAATGATCTTGCTCGTGCTTCGA[THF]CGAGGATGTAGCTGCT-C3 Spacer	47	RPA-LFS	2228035..2228178	144
bla_OXA-51-_R2B	Biotin-AAATACTTCTGTGGTGGTTGCCTTATGGTGC	31			
bla_OXA-51-_F3	GTTATCCAACAAGGCCAAACTCAACAAAGCT	31			
bla_OXA-23-_P	FITC-CTTGTACAATCTGACTCGAGGTTTCATTTA[THF]TACACTATGCTGTGC-C3 Spacer	45		645..910	266
bla_OXA-23-_R2B	Biotin-TTTACTTGCTATGTGGTTGCTTCTCTTTTTCTTTC	35			
bla_OXA-23-_F3	TGGTTCTCCAATCCGATCAGGGCATTCAACATT	33			
bla_OXA-51_-qF	GCAACCACCACAGAAG	16	qPCR	2228158..2228292	135
bla_OXA-51_-qR	TCCAATACGACGAGCT	16			
bla_OXA-23_-qF	CCTGATAGACTGGGACT	16		526..661	136
bla_OXA-23_-qR	ATCGGATTGGAGAACC	17			

F, forward primer; R, reverse primer; P, probe.

### Suitability of the RPA-LFS Assay on *bla*_OXA-51_ and *bla*_OXA-23_

**Figure 2:**
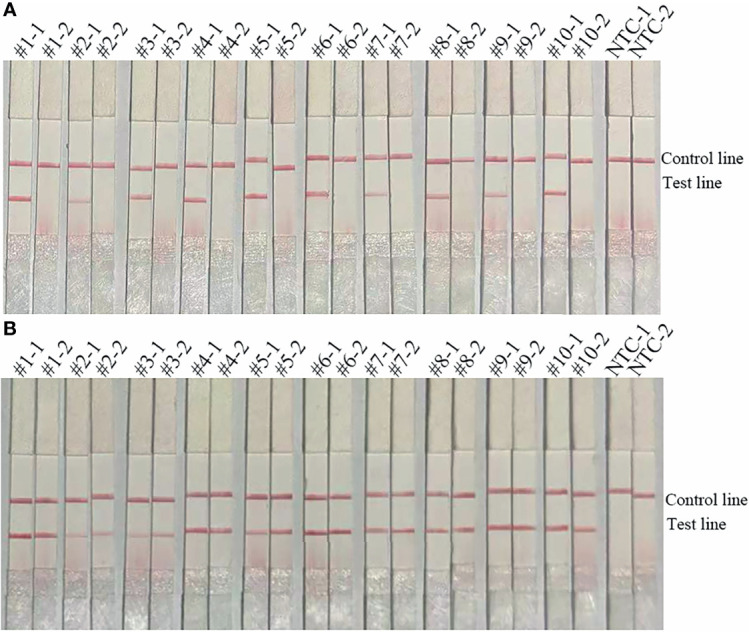
Applicability of the primer-probe sets. **(A)** The image shows the detection results of the RPA-LFS assay for 10 A. *baumannii* (*bla*_OXA-51 without OXA-23_) isolates using the primer-probe sets *bla*_OXA-51_-F3/R2B/P (#1-1, #2-1, #3-1, #4-1, #5-1, #6-1, #7-1, #8-1, #9-1, #10-1) and *bla*_OXA-23_-F3/R2B/P (#1-2, #2-2, #3-2, #4-2, #5-2, #6-2, #7-2, #8-2, #9-2, #10-2). **(B)** The image shows the detection results of the RPA-LFS assay for 10 A. *baumannii* (*bla*_OXA-51 and OXA-23_) isolates using the primer-probe sets *bla*_OXA-51_-F3/R2B/P (#1-1, #2-1, #3-1, #4-1, #5-1, #6-1, #7-1, #8-1, #9-1, #10-1) and *bla*_OXA-23_-F3/R2B/P (#1-2, #2-2, #3-2, #4-2, #5-2, #6-2, #7-2, #8-2, #9-2, #10-2). NTC-1, no template control with the primer-probe set *bla*_OXA-51_-F3/R2B/P. NTC-2, no template control with the primer-probe set *bla*_OXA-23_-F3/R2B/P. The name of each set is shown at the top of each lane. The positions of the test and control lines are indicated on the right. All reactions were performed at 37°C for 30 min. The images represent the results of three independent experiments.

The use of the two primer-probe sets (*bla*_OXA-51_-F3/P/R2B and *bla*_OXA-23_-F3/P/R2B) to detect *A. baumannii* (*bla*_OXA-51 without OXA-23_) demonstrated that the primer-probe set targeting the *bla*_OXA-23_ gene did not yield a positive signal, while only the primer-probe set targeting the *bla*_OXA-51_ gene obtained a positive signal and the NTC did not yield a false-positive signal (**Figure 2A**). Then, the primer-probe sets *bla*_OXA-51_-F3/P/R2B and *bla*_OXA-23_-F3/P/R2B were used to detect the 10 A*. baumannii* (*bla*_OXA-51 and OXA-23_) isolates. All RPA-LFS reactions yielded positive signals without NTC. These results indicate that the primer-probe sets *bla*_OXA-51_-F3/P/R2B and *bla*_OXA-23_-F3/P/R2B can effectively distinguish strains coding for the *bla*_OXA-51_ and *bla*_OXA-23_ genes, respectively (**Figure 2B**). The suitability of the primer-probe sets used in this study was deemed good.

### Specificity of the RPA-LFS Assay for Detection of *bla*_OXA-51_

**Figure 3:**
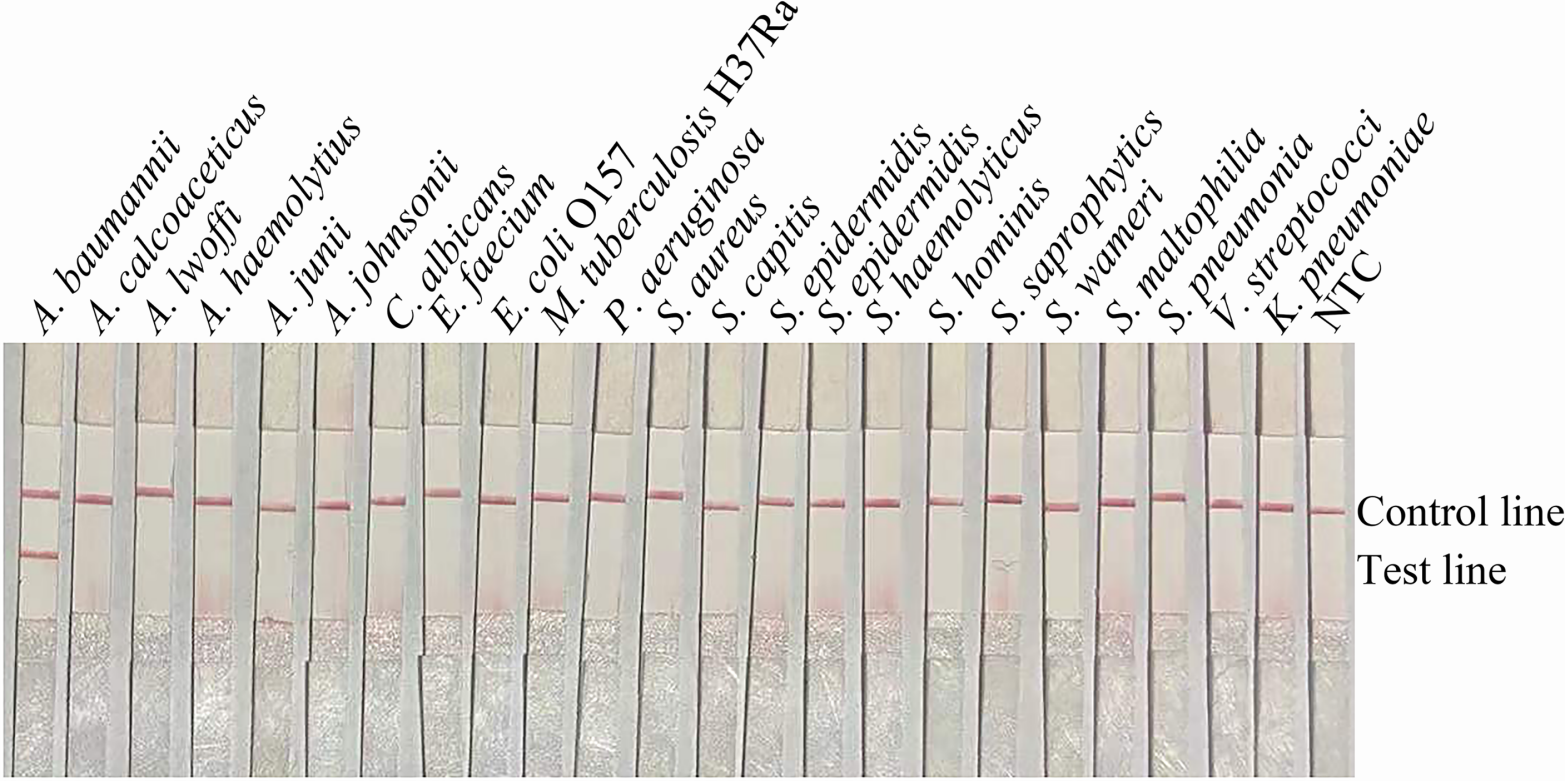
Specificity of the primer-probe sets. The image shows the detection results of the RPA-LFS assay with different bacterial templates using the primer-probe set *bla*_OXA-51_-F3/R2B/P. The name of the bacterium used for each reaction is shown at the top of each lane. NTC, no template control. The positions of the test and control lines are indicated on the right. All reactions were performed at 37°C for 30 min. The image represents the results of three independent experiments.

The primer-probe set *bla*_OXA-51_-F3/P/R2B showed good specificity (**Figure 3**). The primer-probe set *bla*_OXA-51_-F3/P/R2B targeting the *bla*_OXA-51_ gene was highly specific and, thus, used throughout the rest of the study.

### Limit of Detection (LOD) of the RPA-LFS Assay for the *bla*_OXA-51_ and *bla*_OXA-23_ Genes

**Figure 4:**
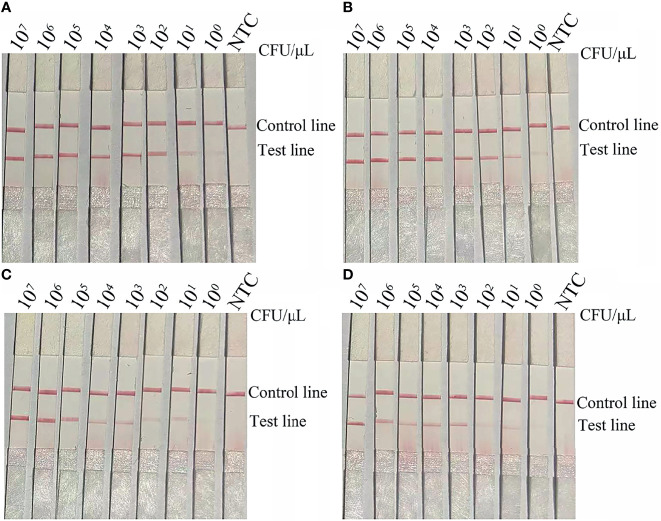
LOD of the RPA-LFS assay. **(A)** The image shows the detection results of the RPA-LFS assay with different CFUs of *A. baumannii* (*bla*_OXA-51_) using the primer-probe set *bla*_OXA-51_-F3/R2B/P. **(B)** The image shows the detection results of the RPA-LFS assay with different CFUs of *A. baumannii* (*bla*_OXA-51_) and 10^7^ CFU of *E. coli* O157 using the primer-probe set *bla*_OXA-51_-F3/R2B/P. **(C)** The image shows the detection results of the RPA-LFS assay with different CFUs of *A. baumannii* (*bla*_OXA-23_) using the primer-probe set *bla*_OXA-23_-F3/R2B/P. **(D)** The image shows the detection results of the RPA-LFS assay with different CFUs of *A. baumannii* (*bla*_OXA-23_) and 10^7^ CFU of *E. coli* O157 using the primer-probe set *bla*_OXA-23_-F3/R2B/P. NTC, no template control. All reactions were performed at 37°C for 30 min. The CFUs are indicated at the top of the strips. The positions of the test and control lines are indicated on the right. The images represent the results of three independent experiments.

The results of the RPA-LFS assay showed that the LOD was 10^0^ CFU per reaction (**Figure 4A**). To mimic conditions of complex contamination, pure *A. baumannii* (*bla*_OXA-51 without OXA-23_) was spiked with 10^7^ CFU/μL of *Acinetobacter lwoffi* and *Escherichia coli* O157. In addition, 10^7^–10^0^ CFU/μL of spiked *A. baumannii* (*bla*_OXA-51 without OXA-23_) were tested with the RPA-LFS assay. The results indicated that the RPA-LFS assay can tolerate interference from other bacteria and the LOD was 10^0^ CFU/μL (**Figure 4B**). Thus, the LOD of the RPA-LFS assay for the *bla*_OXA-51_ gene was 10^0^ CFU. Then, the LOD of the RPA-LFS assay for *A. baumannii* (*bla*_OXA-51 and OXA-23_) was tested using the primer-probe set *bla*_OXA-23_-F3/P/R2B against 10^7^–10^0^ CFU/μL of *A. baumannii* (*bla*_OXA-51 and OXA-23_) (1 μL for each reaction). The results showed that the LOD was 10^1^ CFU per reaction (**Figure 4C**). In addition, the LOD of *A. baumannii* (*bla*_OXA-51 and OXA-23_) spiked with 10^7^ CFU/μL of *A. lwoffi* and *E. coli* O157 was also 10^1^ CFU/μL (**Figure 4D**).

### Application of the RPA-LFS Assay for Detection of the *bla*_OXA-51_ and *bla*_OXA-23_ Genes in Clinical Samples

To mimic an actual application, the RPA-LFS assay was evaluated with 213 clinical samples. All samples were tested for the *bla*_OXA-51_ and *bla*_OXA-23_ genes with the RPA-LFS assay and compared with qPCR. The detection results of RPA-LFS were inconsistent with those of qPCR. In addition, the results showed that the detection rate of *bla*_OXA-23_ was 44.6% (**Table 3**).

**Table 3:** Prevalence of carbapenemase genes in 213 clinical samples of *A. baumannii* using RPA-LFS and PCR (summarized).

Method	*bla*_OXA-51_	N (%)	*bla*_OXA-23_	N (%)
RPA-LFS	206	96.7	95	44.6
qPCR	206	96.7	95	44.6
Coincidence rate (%)	N/A	100%	N/A	100%

N, number.

## Discussion

CRAB poses a serious threat to hospitalized patients worldwide. CRAB infections in hospitals can cause highly mortality (Tekin et al., 2014; Shaheen et al., 2017). Thus, rapid and sensitive diagnosis of CRAB in the early stage of infection is important to ensure patient safety. Current detection methods, including PCR, qPCR, and LAMP, require specific equipment that is not readily available in smaller hospitals. In addition, long periods are required to obtain the results with these methods.

Molecular detection technologies require the selection of a diagnostic amplification target for effective detection of a particular species. Many studies have evaluated various methods for detection of *A. baumannii* infection in sputum samples. With these methods, the *bla*_OXA-51_ gene is most often used as the detection target (Abhari et al., 2021). Although specific to *A. baumannii*, the *bla*_OXA-51_ gene cannot be used to identify CRAB. Hence, the *bla*_OXA-23_ gene has been reported as a reliable target for detection of CRAB (Tekin et al., 2014). Therefore, two primer-probe sets were designed to detect *A. baumannii* and CRAB.

The results of this study indicated that base modifications had no obvious effects on the LOD and the RPA-LFS assay accurately detected *A. baumannii* and CRAB. The LOD of the RPA-LFS assay was 10^0^ CFU for *A*. *baumannii* and 10^1^ CFU for CRAB. This sensitivity was the same as that of the real-time RPA method, which was in the range of 10^0^–10^1^ CFU per reaction (Liu et al., 2020). In addition, the RPA-LFS assay for detection of CRAB was simple and fast, as detection can be completed within 40 min (30 min for amplification and 10 min for LFS analysis). This method requires an isothermal temperature of 37°C, which can be achieved by heating with the hands. The detection results can be easily read without instrumentation in accordance with relatively simple instructions. In contrast, the PCR, qPCR, and LAMP methods require temperature control equipment and relatively long periods for the reaction. The real-time RPA method requires a shorter time than PCR, but requires the use of a fluorescence detector. The total cost of real-time RPA is higher than that of the RPA-LFS assay.

Evaluation of clinical samples showed that the accuracy of the RPA-LFS assay was good. Testing of samples from different patients showed that detection of positive samples with the RPA-LFS was equal to that of qPCR, indicating that the RPA-LFS assay presents an alternative detection method. In addition, the two LFSs used to detect *A. baumannii* and CRAB can be combined into one LFS in the future, which will reduce the cost.

In conclusion, the established RPA-LFS assay is simple, rapid, and accurate, does not require a laboratory facility, and can be combined with a simple and fast DNA extraction method (heat boiling) for home detection of CRAB. Timely diagnosis can facilitate early treatment of nosocomial *A. baumannii* infections.

## Data Availability Statement

The original contributions presented in the study are included in the article/supplementary material. Further inquiries can be directed to the corresponding authors.

## Author Contributions

LW, GH, and XG designed the experiments and wrote the manuscript. YW, DS, and PZ collected the clinical samples. FW, DS, LC, KW, and LW performed the main experiments. YL, XL, and LW analyzed the data. All authors reviewed and approved the final version of the manuscript. All authors contributed to the article and approved the submitted version.

## Funding

This study was supported by grants from the High-level Innovation and Entrepreneurship Talents Introduction Program of Jiangsu Province of China (grant number 2019-30345), the “521 Project” scientific research funding project of Lianyungang City (grant number LYG06521202157), the “HaiYan Plan” scientific research funding project of Lianyungang City (grant number 2019-QD-008), and the Clinical Medical Science and Technology Development Fund of Jiangsu University (grant number JLY20180020).

## Conflict of Interest

The authors declare that the research was conducted in the absence of any commercial or financial relationships that could be construed as a potential conflict of interest.

## Publisher’s Note

All claims expressed in this article are solely those of the authors and do not necessarily represent those of their affiliated organizations, or those of the publisher, the editors and the reviewers. Any product that may be evaluated in this article, or claim that may be made by its manufacturer, is not guaranteed or endorsed by the publisher.
